# Odour source distance is predictable from a time history of odour statistics for large scale outdoor plumes

**DOI:** 10.1098/rsif.2024.0169

**Published:** 2024-07-31

**Authors:** Arunava Nag, Floris van Breugel

**Affiliations:** ^1^ Computer Science Engineering Department, University of Nevada, Reno, NV, USA; ^2^ Integrative Neuroscience Program, University of Nevada, Reno, NV, USA; ^3^ Ecology Evolution and Conservation Biology Program, University of Nevada, Reno, NV, USA; ^4^ Department of Mechanical Engineering, University of Nevada, Reno, NV, USA

**Keywords:** odour plume statistics, olfactory navigation, turbulent plumes

## Abstract

Odour plumes in turbulent environments are intermittent and sparse. Laboratory-scaled experiments suggest that information about the source distance may be encoded in odour signal statistics, yet it is unclear whether useful and continuous distance estimates can be made under real-world flow conditions. Here, we analyse odour signals from outdoor experiments with a sensor moving across large spatial scales in desert and forest environments to show that odour signal statistics can yield useful estimates of distance. We show that achieving accurate estimates of distance requires integrating statistics from 5 to 10 s, with a high temporal encoding of the olfactory signal of at least 20 Hz. By combining distance estimates from a linear model with wind-relative motion dynamics, we achieved source distance estimates in a 60 × 60 m^2^ search area with median errors of 3–8 m, a distance at which point odour sources are often within visual range for animals such as mosquitoes.

## 1. Introduction

Odour plumes serve as a crucial sensory signal for many organisms searching for food and mates [1], as well as robotic systems geared towards odour source localization [2,3]. In outdoor environments, these plumes are transported by turbulent air currents, which break them into complex, intermittent, and time-varying patterns [4–7]. As a result, a single encounter with an odour plume does not provide direct information about how far away an odour source is. Despite the challenges imposed by this structure, a variety of organisms, including insects [8–10], crustaceans [11], fish [12] and mammals [13], excel at following intermittent odour plumes towards their source. Their success has long served as motivation and inspiration for engineers aiming to develop similarly capable algorithms for applications such as search and rescue and environmental monitoring [14–16]. How animals and robots alike can solve the challenging task of plume tracking fundamentally depends on the information they can extract from their experience, and in particular, under natural wind conditions. Here, we address a fundamental question in the field of odour plume dynamics and navigation: can source distance information realistically be extracted from odour plume statistics for an agent moving across large spatial scales under natural wind conditions?

Historically, the prevailing dogma has been that animals primarily respond to their immediate olfactory experience in a reactive manner by surging up-wind after an odour encounter and casting cross-wind after losing the plume [8], and many algorithms for robotic systems have taken a similar approach [17–20]. However, subsequent studies have shown that animals do modulate plume tracking decisions based on a time history of odour encounters [21,22]. Furthermore, animals qualitatively change their behaviour when they get close to the source, including flying insects [23], which approach visual features when they get close [8,24], and dogs [25] that modulate their search speed (see also [26] for a review). These observations suggest that animals are able to extract information about source distance from their olfactory experience, either in the form of a true distance estimate or through a simple correlative heuristic. Whether or not this is possible, and which statistical features of the olfactory experience are most informative, especially over large spatial scales with natural outdoor flow conditions, is not well understood.

To describe the statistics of intermittent odour plumes, we use the established term *whiff* to indicate a collection of odour encounters above some threshold (also referred to as a *bout*), and the term *blank* for anything below that threshold [7,27]. Prior experiments suggest that both the intensity of whiffs (i.e. concentration) and their timing (e.g. whiff frequency, wf) are correlated with distance. Celani *et al.* [7] showed agreement between theory and both lab and field experiments (from [28]) that the probability distributions of whiff frequencies and concentrations vary with distance for a stationary sensor. Additional experiments on spatial scales of a metre or so using experimental and computational approaches and stationary sensors have further confirmed that both timing and intensity of odour whiffs carry information correlated with distance to the source [27,29–31]. Across these studies researchers have used a diversity of statistics to characterize whiff structure, including: mean odour concentration, wf, average slope of whiffs, average duration of blanks and whiffs, intermittency factor, and variance of wf. Many of these statistics do modulate olfactory navigation behaviors of animals such as insects: concentration modulates the probability of turning up-wind in flying flies [21], and odour encounter frequency and other statistics drive olfactory navigation decisions in walking flies [32–36]. What remains unknown, is the feasibility of distinguishing between distributions of whiff statistics from a finite amount of time in natural environments, where wind can be quite variable in both speed and direction [37]. In this paper, we tested whether the timing and concentration features of an odour signal can be used to estimate distance from the source in outdoor environments at spatial scales of 60 × 60 m^2^. We found that features of individual whiffs have a very weak correlation with distance from the source. However, by accumulating odour signal statistics—specifically whiff concentration and duration—over a 10 s time window from a moving sensor (thus capturing both spatial and temporal information) it is possible to estimate the distance to an odour source with surprising accuracy, especially under consistent wind conditions. However, this only works well if odour information is encoded at a relatively high temporal frequency—with a low-pass filter cut-off of 20 Hz or more. Intriguingly, our findings for both the encoding frequency and the temporal integration time are consistent with established observations for model plume tracking organisms such as the fruit fly.

## 2. Results

### 2.1. Outdoor measurements of odour plumes

**Figure 1.**
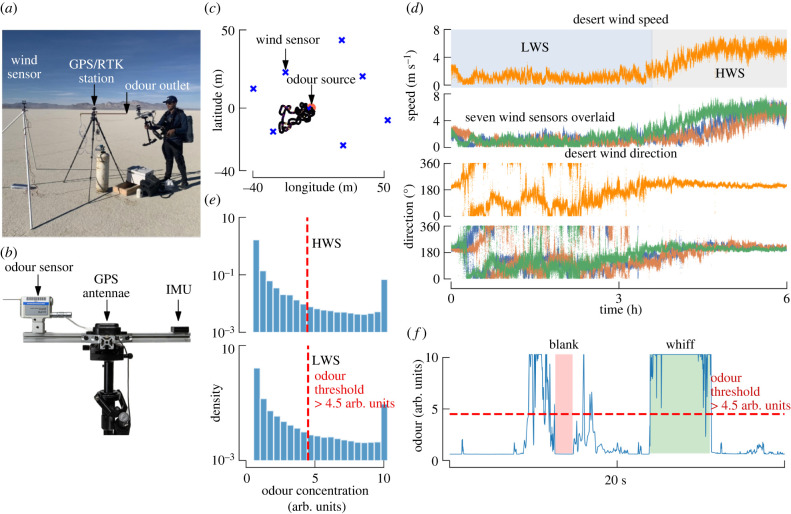
Experiment design: desert playa. (*a*) Image from our Black Rock Desert field site showing the propylene odour source, GPS RTK antennae, one of eight ultrasonic anemometers, and gimbal supported sensor unit. (*b*) Mobile sensor unit including a mini photo-ionization detector, GPS antenna and IMU. (*c*) Representative portion of our walking trajectory shown in relation to the odour source and anemometers. (*d*) Wind speed and direction as a function of time for the duration of the 6 h experiment for the anemometer closest to the odour source (orange traces), and all other anemometers (other colours). In our subsequent analysis, we divided the data collected from this field site into two separate scenarios corresponding to the period characterized by low wind speeds and high directional variability (blue shading—HWS), and high wind speeds and low directional variability (grey shading—LWS). (*e*) Log-scaled histogram of odour concentration recordings for this field site. (*f*) Representative time trace of the odour signal, illustrating the intermittent nature characterized by whiffs (contiguous odour encounters greater than the 4.5 arb. units threshold) and blanks (times when the odour signal was below the threshold). More such odour traces can be seen in electronic supplementary material, figure S3.

To investigate the correlation between odour signal statistics and distance to the source, we collected data in two environments. First, we selected a flat desert playa in Northern Nevada’s Black Rock Desert (figure 1*a*). We used a propylene tracer gas as an odour source, emitted from a 0.014 m diameter copper tube 2 m above the ground at a pressure of 10 PSI. We developed a mobile sensor stack consisting of a photo-ionization detector to record odour concentration, a GPS real time kinematics (RTK) receiver to record position, and an inertial measurement unit (IMU) to measure angular velocity (figure 1*b*). We placed eight wind sensors around the odour source to measure the ambient wind direction and speed, and traversed this 60 × 60 m^2^ space by foot while carrying the sensor stack for 6 h (figure 1*c*).

The horizontal wind speed and direction were spatially consistent throughout the duration of the experiment (figure 1*d*), with comparatively low magnitudes of vertical wind (electronic supplementary material, figure S1*a*). In subsequent analyses, we used the closest stationary wind sensor to the odour source to represent the ambient wind speed and direction. Over the course of the day, the wind transitioned from low speeds and high directional variability to a strong southerly flow, indicating a shift in the local wind regime. We split our data into two datasets accordingly: ‘low wind speed’ (LWS) less than 3.5 m s^−1^ and ‘high wind speed’ (HWS) greater than 3.5 m s^−1^ (figure 1*d*).

**Figure 2.**
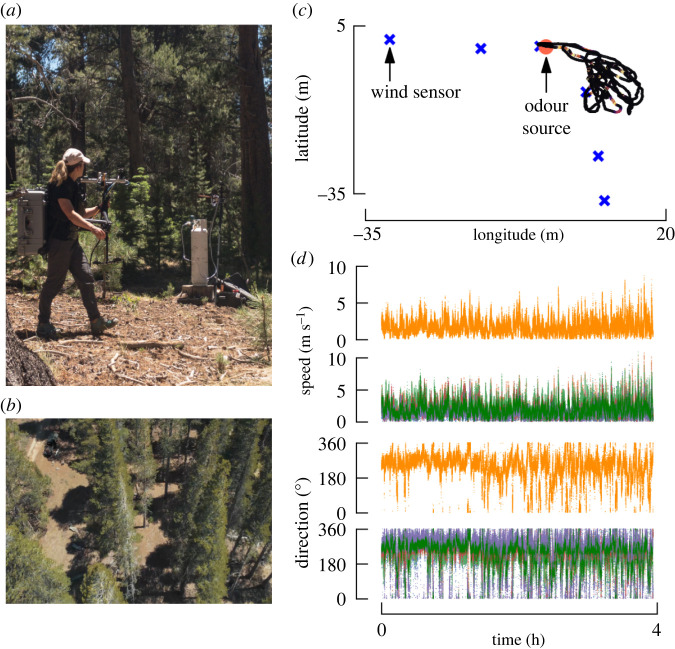
Experimental design: forest. (*a*) We collected data by traversing the area surrounding the odour source on foot. (*b*) Aerial view of the field site illustrating the tree density. (*c*) Representative portion of our walking trajectory around the odour source, and arrangement of the ultrasonic anemometers. (*d*) Wind speed and direction as a function of time for the anemometer closest to the odour source (orange traces), and all other anemometers.

For our second environment, we chose the Whittell Forest along Eastern Sierra Front in Nevada (figure 2*a*,*b*). We used a similar experimental arrangement but with the wind sensors positioned in an L-shape (figure 2*c*). Although in this environment, we did find a higher degree of spatial variability of the wind, overall trends were similar across wind sensors, and we again chose the closest wind sensor as a reference for the ambient wind speed and direction (figure 2*d*). Again, vertical wind speed was comparatively low (electronic supplementary material, figure S1*b*).

The high variability in wind direction in our LWS and forest environments would have resulted in very few encounters with the odour plume if we had used a stationary odour sensor or one guided by entirely random movements. Instead, we used our real-time odour concentration signal to drive audio cues allowing us to centre our walking trajectory around the plume to efficiently gather data about as many individual odour whiffs as possible. We attempted to follow trajectories reminiscent of the casting motifs exhibited by flying insects [8]. The majority of our movement was in the plane horizontal to the ground at walking speeds of approximately 1–2 m s^−1^. Our changes in direction in line with the source, and perpendicular to the source, ranged from 0.02–0.2 Hz (electronic supplementary material, figure S2). For comparison, flies and moths cast at about 0.5−1 Hz = 1 −2 turns s^−1^ [8,38,39]. We made small vertical movements on the scale of ±0.5 m, generally staying aligned with the altitude of the source. Encounters with odours were infrequent: most of the time, our miniature portable photo-ionization detector (miniPID) recorded values below 2 arb. units, with occasional spikes in the readings (figure 1*e*). The miniPID sensor recorded measurements between 2 and 4 arb. units in the field when testing only ambient wind or human breath. We established a threshold of 4.5 arb. units (2.2 times the standard deviation [34] (*σ*) of all odour measurements) to categorize odour signals as either *whiffs* or *blanks* (figure 1*f*) to minimize ambient noise interference, drawing inspiration from the techniques used in the study by Jayaram *et al.* [34]. We also performed a sensitivity analysis to quantify the impact of different thresholds on our overall conclusions (electronic supplementary material, figure S4).

We made our outdoor plume measurements on 2 days that were a part of our larger survey of near surface wind characteristics [37]. The ambient wind conditions for our odour plume experiments spanned close to the full range of typical conditions that we observed across 10 days of wind measurements in playa, sage steppe and forest environments (electronic supplementary material, figure S5). Altogether, our plume measurements from the desert and forest experiments spanned 10 h of active data collection resulting in a total of 11 107 whiffs (see electronic supplementary material, table S1 for breakdown across scenarios).

### 2.2. Distribution of odour whiff locations is driven by wind characteristics

**Figure 3.**
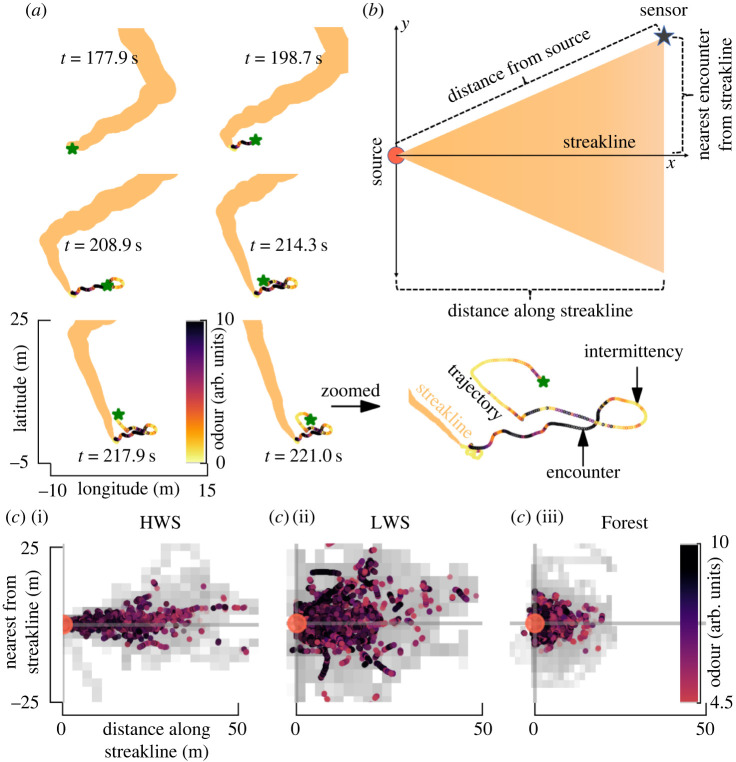
Streakline-aligned odour encounters reveal approximate shape and extent of the outdoor odour plumes. (*a*) Six snapshots spanning 43 s showing the ideal streakline (orange, determined by integrating the ambient wind vector), together with our walking trajectory colour-coded by the odour concentration recorded at each location. Green star indicates the location of the sensor for the same moment that the streakline is shown. The width of the streakline is for illustration purposes, not drawn to scale. (*b*) To visualize the extent of the plumes despite the time varying wind direction, we transformed each point along the trajectory from latitude/longitude to streakline coordinates. We calculated the distance from the sensor to the source and the shortest distance to the streakline. (*c*) Using the approach described in *b*, we transformed each whiff (i.e. when the odour signal was greater than 4.5 arb. units) into streakline coordinates for each of the three scenarios (i–iii). Grey shading indicates time spent in streakline coordinate space, where darker shade indicates time spent more than 10 s. For a more clear histogram of time spent, please refer to electronic supplementary material, figure S6. *N* = 2842, 3522, 4986 whiffs for HWS, LWS and Forest, respectively.

Before analysing the correlation between odour statistics and distance to the source, we wanted to visualize the shape of the odour plumes in each condition. Because the wind direction varied throughout the course of our experiments, we developed an approach to ‘straighten’ the data by aligning encounters relative to an ideal streakline. We determined the ideal streakline by integrating the wind velocity vectors over time. Figure 3*a* shows six snapshots of this moving streakline evolving over time. For each moment in time, we determined the shortest distance from the sensor location to the streakline centreline, and the distance from the sensor to the source. These two values allowed us to project sensor locations into a new streakline-centred coordinate frame (figure 3*b*).

Figure 3*c* shows the streakline-centred locations where we encountered odours for all three scenarios. In the HWS dataset from the desert playa, the plume was quite narrow with odour encounters occurring close to the ideal streakline, and becoming rare at distances of 25 m away (figure 3*c*_*i*_). For the LWS dataset, odour encounters were spread out away from the streakline centre much more widely (figure 3*c*_*ii*_). For our Forest dataset (figure 3*c*_*iii*_), odour encounters were mostly found within a smaller 10–15 m radius from the source and did not have the same cone-like shape seen in the desert datasets. This could be because the wind was more variable due to the trees spread throughout the area. In each case, examples of both low- and high-concentration encounters can be found across the extent of the plume, though high-concentration encounters were more frequent closer to the source.

### 2.3. Odour whiff statistics for estimating distance to source

**Figure 4.**
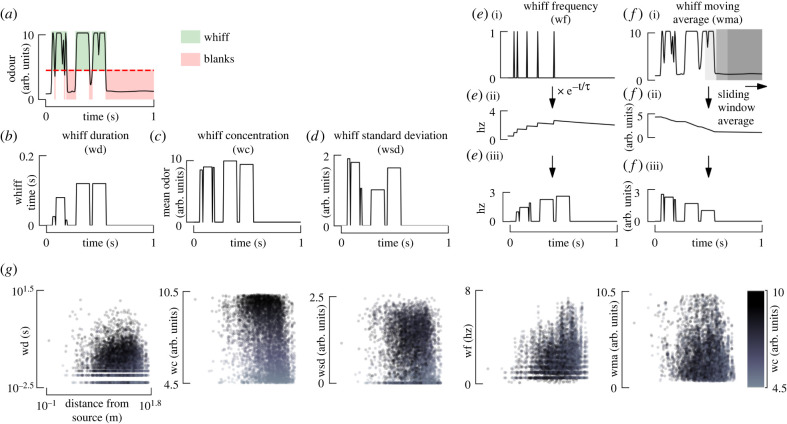
Individual whiff statistics are not strongly correlated with distance. (*a*) Representative trace of odour concentration over time, colour-coded according to whiffs and blanks. (*b*–*d*) For each individual whiff, we calculate a single value corresponding to the whiff duration (wd), mean whiff concentration (wc) and standard deviation of the concentration within a whiff (wsd), resulting in piecewise constant time traces. (*e*) Whiff frequency (wf) is calculated by convolving an exponential decay kernel (*τ* = 2) with a binary delta function corresponding to the onset of each whiff. Each whiff is assigned a single value for the whiff frequency. (*f*) We calculated the moving average of odour concentration using a sliding window (1 s), and then assigned each whiff a single value corresponding to the mean of the moving average signal during the time frame of the whiff (wma). (*g*) Statistics of individual whiffs are not strongly correlated with distance to source. Each panel shows a scatter plot of the whiff statistics defined above for all three scenarios combined. Colour encodes the whiff concentration. Whiff statistics for the three scenarios are shown separately in electronic supplementary material, figure S7. *N* = 2842, 3522, 4986 whiffs for HWS, LWS, and Forest, respectively.

To test if odour signal statistics are correlated with distance from the source, we calculated the following odour statistics (figure 4): whiff duration (wd), mean concentration during a whiff (wc), wf (following calculations from [32], see Material and methods), whiff moving average (wma) and whiff standard deviation (wsd) of the encounters during a whiff. We define a single (average) value for each statistic for each individual whiff, resulting in the stepwise constant plots shown in figure 4*b*–*f*.

### 2.4. Individual whiff statistics are not strongly correlated with distance

To identify potential correlations between our selection of odour statistics and distance from the source, we constructed a multiple linear regression model for each of the three scenarios (table 1; see electronic supplementary material, figure S8 for residual analysis). We found whiff concentration and frequency to be the most significant statistics, but both had very subtle correlations with distance. Because we collected data by moving the sensor in a biased manner, we also considered the effects of our motion relative to the instantaneous wind direction, but did not find any meaningful difference in the results (electronic supplementary material, table S2).

**Table 1.** *R^2^* and semi-partial *r* for distance correlation with ∼ odour statistics.

		semi-partial *r* (% contribution)				
dataset	*R*^2^	wc	wf	wd	wma	wsd
HWS	0.14	44.03^**^	29.17^**^	5.02*	3.41*	5.45^**^
LWS	0.13	70.53^**^	7.98^**^	26.72^**^	0.03*	0.48*
Forest	0.08	81.29^**^	1.97*	22.10^**^	1.21*	0.21*
combined	0.13	89.44^**^	10.03*	24.87^**^	1.43*	1.37*

*Note*: *for 1 × 10^−5^ < *p* < 0.05, ^**^for *p* < 1 × 10^−5^.

Although we found individual whiff statistics to be poorly correlated with distance, the distributions of these statistics (figure 4*g*) do exhibit consistent patterns in relation to distance. For example, whereas concentration of an individual whiff might be high, or low, across a wide range of distances, the distribution of whiff concentrations clearly trends to smaller values at higher distances. At larger distances, whiff frequencies were more likely to be higher, thus high-frequency but low-concentration sequences of whiffs should be expected at large distances. At shorter distances, whiffs were rare (low whiff frequency), but when they did occur, they had a relatively long whiff duration and high wma. Finally, the overall pattern of the wsd bears some resemblance to the whiff concentration, a relationship we explore more later. In the next section, we examine how the distribution of a time history of whiffs is correlated with distance.

### 2.5. A short time history of whiff statistics reveals strong correlations with distance

**Figure 5.**
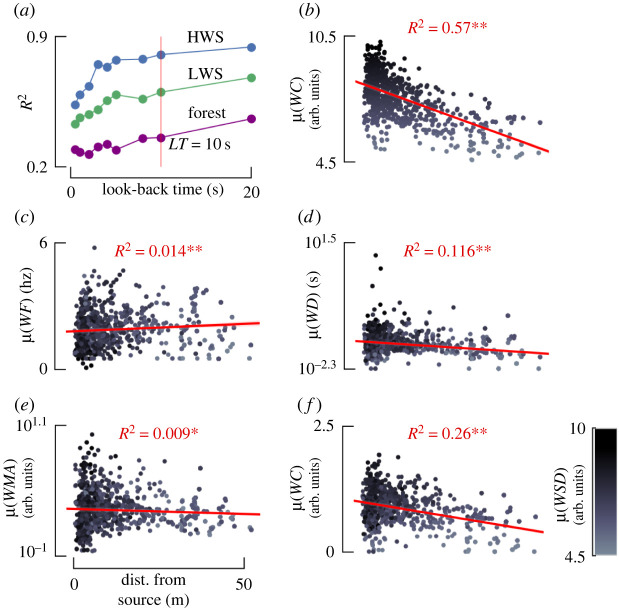
Meta statistics for whiffs occurring within a 10 s ‘look-back’ time window are strongly correlated with distance from the source. (*a*) For the collection of whiffs occurring within a chosen look-back time for randomly sampled trajectory snippets (see text), we calculated five meta statistics: mean, standard deviation, min, max and kurtosis of each of our whiff statistics. The correlation coefficient for a linear regression relating these 25 meta statistics to the source distance rises with increasing look-back times. In subsequent analyses, we chose to focus on a look-back time of 10 s. (*b*–*f*) Distribution of the mean of each whiff statistic with respect to the distance from the source for a look-back time of 10 s. Here, data are combined across all three scenarios. Red line shows the correlation. See electronic supplementary material, table S3 for detail.

In this section, instead of considering individual whiff statistics, we collect all the whiffs that occurred within a short ‘lookback’ time window (i.e. a trajectory snippet, see Material and methods). This analysis results in a (sparse) probability distribution associated with each whiff statistic for a given distance (the average distance for that trajectory snippet). To determine whether these probability distributions are correlated with distance we quantified these distributions using five meta statistics: mean (*μ*), standard deviation (*σ*), *min*, *max* and kurtosis (*k*). Applying these five meta statistics to our five whiff statistics results in 25 features. We first looked for correlations between these 25 features and distance to the source for a range of look-back times. We found a sharp increase in *R*^2^ for look-back times from 1 to 5 s, with a continued but modest increase for look-back times up to 10 s resulting in final *R*^2^ values at 10 s ranging from 0.38 to 0.82 (figure 5*a* and table 2). Figure 5*b*–*f* shows how the mean of the five whiff statistics are correlated with distance, revealing that the mean of whiff concentration and mean of the standard deviation of each whiff both exhibit significant and strongly predictive correlations (electronic supplementary material, table S3). Electronic supplementary material, figure S4 shows a sensitivity analysis of these correlations to the choice of odour threshold. Across a range of thresholds, the mean of whiff concentration and the mean of wsd consistently show the highest correlation with source distance across all three scenarios.

**Table 2.** *R^2^* and model significance (*p*-value) correlating distance from the source with all 25 meta whiff statistics collected over a look-back window = 10 s.

dataset	*R*^2^	*f* −*p*-value
HWS	0.82	extremely small
LWS	0.61	8.94 × 10^−226^
Forest	0.38	8.77 × 10^−65^
all combined	0.65	1.67 × 10^−300^

### 2.6. Concentration and timing meta statistics are correlated with source distance

Next, we set out to understand which meta statistics of our whiff statistics provide the most predictive power for distance from the source. We chose to focus on a look-back time of 10 s for the rest of our analysis, as beyond this point *R*^2^ does not substantially improve. We combined datasets from desert and forest environments and individually correlated the 25 meta statistics with distance (electronic supplementary material, figure S10). To compare the relative quality of different models and avoid overfitting we used Akaike’s information criterion (AIC) [40] to construct a series of statistical models with increasing complexity (electronic supplementary material, table S4). AIC penalizes models with more parameters, while rewarding those that fit the data well. The single most informative meta statistic was the mean whiff concentration. Small improvements in *R*^2^ and AIC were realized as more meta statistics were included in the model, but after approximately four meta statistics, the changes in AIC (Δ*AIC*) resulted in diminishing returns. With four parameters, the lowest AIC identified *μ*(*WC*), *σ*(*WD*), *max*(*WMA*) and *σ*(*WMA*) as the most informative meta statistics for estimating distance.

Notably missing from the list of meta statistics selected by our AIC analysis is *μ*(*WSD*), which is strongly correlated with distance (figure 5*f*). This is because *μ*(*WC*) and *μ*(*WSD*) are mutually correlated (electronic supplementary material, figure S9, *R*^2^ = 0.51, *p* = 4.61 × 10^−228^), so including *μ*(*WSD*) in a statistical model that already contains *μ*(*WC*) does not provide enough novel information and would violate model assumptions, increase bias and skew the regression results. The strong correlation between *μ*(*WC*) and *μ*(*WSD*) suggests that these two meta statistics are somewhat interchangeable.

**Figure 6.**
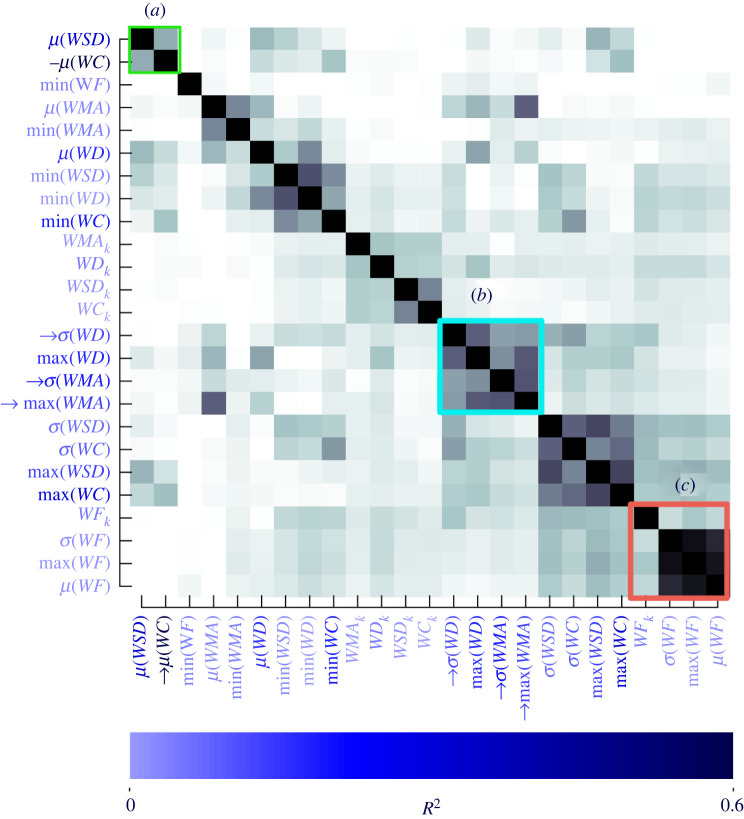
Hierarchical clustering reveals distinct clusters of mutually related meta statistics of whiffs within a look-back window. Darker colours in the adjacency matrix correspond to higher pairwise correlations between meta statistics. The *R*^2^ correlation coefficient between each individual meta statistic and distance to the source is encoded in the blue shading of the meta statistic labels. Arrows indicate meta statistics selected by the four-parameter model from electronic supplementary material, table S4. (*a*) Concentration related parameters *μ*(*WC*) and *μ*(*WSD*) are mutually correlated (see also electronic supplementary material, figure S9), and are both strongly correlated with the source distance (see also electronic supplementary material, figure S10*a*). (*b*) The parameters related to whiff duration—*σ*(*WD*), *max*(*WMA*), and *σ*(*WMA*)—form a distinct group, three of which are also selected by the AIC analysis. (*c*) Whiff frequency meta statistics form a cluster and provide unique information about the distance to the odour source, albeit low correlation with distance to the source.

To compactly visualize all strongly correlated groups of meta statistics, we constructed a hierarchically clustered matrix of the correlations between each pair of meta statistics (figure 6). The resulting adjacency matrix identifies *μ*(*WC*) and *μ*(*WSD*) as their own cluster (figure 6*a*), suggesting that these interchangeable statistics provide unique information compared with other whiff statistics, and that the mean and variability (*WC* and *WSD*, respectively) within a whiff are related. Both meta statistics decrease with increasing distance (figure 5*b*,*f*), perhaps because at larger distances diffusion has had a chance to both even out the odour distribution and reduce the concentration within a whiff. Overall this cluster can be summarized as one that captures concentration information.

Our AIC analysis also identified *σ*(*WD*), max(WMA) and *σ*(*WMA*) as informative, and we find that these whiff statistics form their own cluster in the adjacency matrix (figure 6*b*). Because longer whiff durations will typically result in larger wma, this cluster can be summarized as one that captures variability in whiff duration (and its maximum), e.g. a measure of odour timing.

The next clear cluster (below and right of (*b*)) includes meta statistics that describe the variability of both whiff concentration and standard deviation across whiffs. Although these meta statistics are correlated with distance, our AIC analysis suggests variability of these concentration related statistics may not be as informative as their mean (note the correlation between this cluster and cluster (*a*)).

Finally, we see that meta statistics of wf form a distinct cluster (figure 6*c*), indicating that wf provides unique odour timing related information compared with our other whiff statistics. However, wf is not strongly correlated with distance in our experiment (electronic supplementary material, table S3, *R*^2^ = 0.014, *p* = 4.0 × 10^−6^). We discuss this apparent conflict with existing literature in the discussion.

### 2.7. Integrating whiff statistics with relative motion can provide a continuous estimate of source distance

The intermittent nature of odour whiffs means that any estimate of distance based solely on whiff statistics will also be highly intermittent and therefore of limited utility. To illustrate how this signal could be used to generate a continuous estimate of distance we used a constant-velocity Kalman smoother to integrate two measurements (see Material and methods).

First, we used our four-parameter statistical model for a look-back time of 10 s (electronic supplementary material, table S4) to generate intermittent distance predictions. We combined the data from all three of our scenarios, and for our odour delivery paradigm across these environments the distance predictions are given by the following equation:

2.1 distance=−8.85μ(WC)+0.20max(WMA)−2.43σ(WMA)−3.07σ(WD)+84.40.

**Figure 7.**
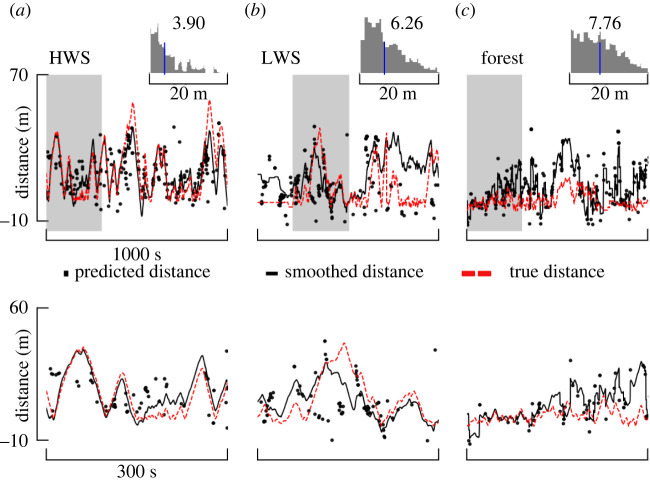
Integrating whiff statistics with relative motion in a Kalman smoother yields continuous estimates of source distance. (*a*–*c,* upper row): comparison of predicted, Kalman-smoothed and true distances for three scenarios. Our Kalman smoother combined intermittent distance estimates from our combined desert + forest linear model with continuous estimates of our ground velocity component parallel to the ambient wind. Histograms show the median error between the Kalman smoothed and true distance. Lower row: a magnified view of the shaded regions from the upper row. For an even zoomed-in presentation of the above-smoothed results and if environment-specific prediction models are used, refer to electronic supplementary material, figure S12.

Second, we used continuous estimates of the sensor ground velocity (acquired via GPS) component parallel to the instantaneous ambient wind direction. Despite the model simplicity, our smoothed continuous distance estimates produced surprisingly accurate distance estimates in both the desert HWS and LWS scenarios, achieving median errors in absolute distance of 3.9 and 6.26 m, respectively (figure 7*a*,*b*). For the Forest scenario, our estimates consistently overestimated the true distance, resulting in a median error of 7.76 m (figure 7*c*). Overall, these results were relatively independent of the choice of odour threshold (electronic supplementary material, figure S4*a*). For the HWS case, a threshold of 4.5 arb. units yielded the minimum error for HWS, however the errors stayed mostly constant for LWS and Forest across thresholds.

Next, we asked how much the statistical models varied across our three scenarios, and whether using environment-specific models might improve the accuracy of distance estimates. Only the intercept of the model changed substantially across the scenarios, dropping consistently with increasing variability in wind direction (electronic supplementary material, figure S11). In other words, equivalent whiff statistics in scenarios with higher wind variability generally correspond to shorter distances. Although incorporating these scenario-specific model parameters does improve the accuracy of distance estimates, the changes in performance are modest (electronic supplementary material, figure S12).

### 2.8. Odour signal temporal resolution needs to be upwards of 20 Hz for accurate distance estimation

**Figure 8.**
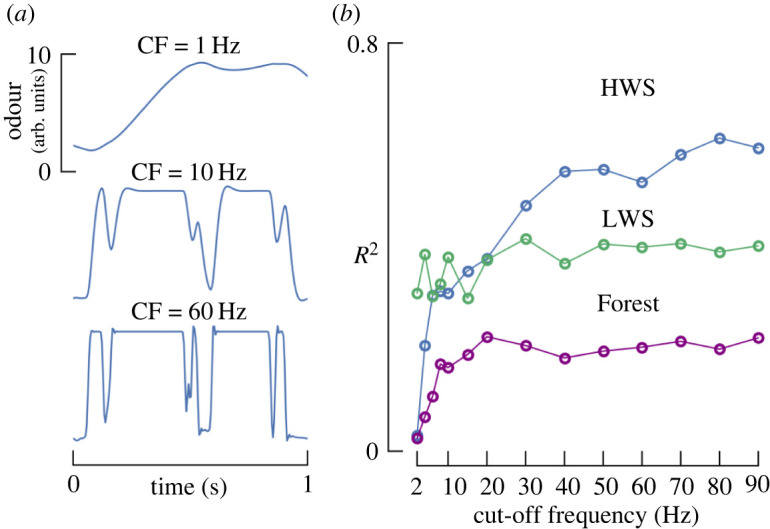
Low-pass filtering odour signal degrades correlations between whiff statistics and source distance. (*a*) Sample time traces of odour signal for selected cut-off frequencies used in a second-order Butterworth filter and varied the cut-off frequency from 1 to 90 Hz (approximately half the Nyquist frequency), while keeping the order of filter constant at two. (*b*) The correlation coefficient for distance correlated with AIC-filtered whiff meta statistics for a look-back time of 10 s as a function of cut-off frequency (CF). Correlation coefficients shown here are smaller than those in figure 5 because this analysis uses only 4 of the 25 parameters, selected by our AIC analysis.

Finally, we asked whether a high temporal resolution of the odour signal is necessary to accurately estimate the distance to the source, or if slower time-averaged measurements would perform equally well. To answer this question, we low-pass filtered our 200 Hz odour signal time series using a second-order Butterworth filter [41]. Figure 8*a* shows representative time traces of the filter’s effects. For each cut-off frequency, we re-ran our analysis pipeline to identify the new whiffs, their statistics, the meta statistics for a 10 s look-back window and calculated the *R*^2^. Our results show that cut-off frequencies of less than 20 Hz generally result in substantially reduced predictive power (especially in the HWS and Forest scenarios) (figure 8*b*).

## 3. Discussion

Our outdoor experiments showed that a moving agent could estimate source distance by integrating a 5–10 s time history of whiff statistics together with their movement relative to the wind. Furthermore, we showed that this is only feasible given a high odour sampling frequency of 20 Hz or more. Consistent with prior work using metre-scale fluid dynamics simulations [27], we found that both concentration features (mean concentration and standard deviation of concentration within a whiff, figure 6*a*), and timing features (whiff duration and moving average, figure 6*b*) provide complementary information. Contrary to wind tunnel experiments [7,31], however, we found that wf formed its own distinct cluster, which was not strongly correlated with distance (figure 6*c*). Only for individual whiffs in consistent wind (our HWS case) was wf more predictive compared with whiff duration, though its utility was still limited (*R*^2^ = 0.04). In our look-back analysis wf did drop to very low values close to the source (possibly driven by either sensor movements or changes in wind direction), suggesting that perhaps a low frequency of encounters could be indicative of approaching the source. Together, our results are consistent with the idea that for a sensor moving slowly relative to wind, wf may carry useful information. However, under more naturalistic conditions that involve either dynamic wind, or a moving sensor that may or may not stay within the odour plume envelope, wf may be an unreliable cue when accumulating evidence over a longer look-back window, and the duration of individual whiffs may be more informative.

Our field experiments were necessarily limited due to the logistics of gathering this type of data. Accordingly, we chose to use a single source geometry, odorant, release concentration, and flow rate for all three scenarios. The specific values of the coefficients relating distance to each of the whiff statistics we considered would probably change for different choices of these parameters. We used a miniPID chemical sensor and used the system’s maximum settings; however, we did not account for scenarios of signal saturation, which could be mitigated in the future by using a sensor with a higher range, or collecting data at a variety of gain settings. Our sensitivity analysis (electronic supplementary material, figure S4), however, suggests that the intermediate threshold we chose for our analyses (4.5 arb. units) was a near optimal choice for this dataset.

Thresholds below 2–4 arb. units led to poorer correlations with source distance (electronic supplementary material, figure S4*b*–*d*), probably because of higher levels of ambient noise, whereas higher thresholds also resulted in lower correlations in many cases, probably by removing too much signal information. The minor improvement in distance estimation accuracy we achieved when considering environment-specific models instead of a single combined model, however, suggests that distance estimation can be relatively robust to specific characteristics of the wind environment (electronic supplementary material, figure S12). The coefficient related to the whiff concentration is probably the one that would change the most for other types of source conditions. Our hierarchical clustering analysis suggests that robustness to differences in release concentrations may be realized by relying on the standard deviation within a whiff more so than the concentration. Alternatively, *a priori* knowledge of the type of source might be needed to achieve reliable distance estimates. In the context of olfactory navigation among animals or machines this could be achieved through niche- or task-specific models. For example, a male moth following a sparse pheromone plume could use a different distance estimation model compared with a mosquito following a CO_2_ plume to a human host, or a fruit fly searching for decaying fruit matter.

Might animals, such as flying insects, use whiff statistics to estimate the distance to a source? The qualitative changes exhibited by flying insects when they get close to a source [23,26] indicate that there is probably at least a correlational mechanism linking their olfactory experience with their behavioural decision to approach visual features [8,24]. In our experiments, we achieved an accuracy of 3–8 m, depending on the environment. This is in line with the range over which insects such as mosquitoes are likely to begin visually resolving potential odour sources [42,43], making it a practically useful estimate for gating such behavioural decisions.

Gating a qualitative behavioural change could be accomplished by an internal estimate of distance, or a simpler binary threshold heuristic. In either case, our results suggest that a working memory of approximately 5–10 s and a sampling frequency of 20 Hz are required. Experiments with flying mosquitoes [42], and flying [8] and walking fruit flies [36] indicate that odour can influence their behaviour on time scales of 10 s. Furthermore, when flies encounter a plume multiple times, they adjust the strength of their up-wind turns, suggesting that they do integrate odour information across multiple encounters on time scales of at least 3 s [21]. Meanwhile, peripheral olfactory receptors in fruit flies are characterized by a low-pass filter frequency of roughly 10–20 Hz [44,45], and arguably even faster [46]. Finally, many of the whiff statistics, we identified are known to modulate aspects of plume tracking behaviour in flies: concentration regulates the strength of flying flies’ up-wind turns [21]; duration has a subtle effect on flying flies’ probability of turning up-wind and approaching visual features [8]; walking flies adjust their decisions based on wf [32]; and flying moths require intermittent odour signals to maintain an up-wind surging flight behaviour [10].

Our field experiments provide clear evidence that meaningful source distance information can be extracted from a time history of odour encounter statistics in outdoor scenarios with natural wind. Future studies with plume tracking animals should focus on answering the question of whether they use the types of statistics we identified to either generate a continuous representation of distance, or drive a binary heuristic. Meanwhile, engineers aiming to develop odour localizing robots should consider incorporating source distance estimation algorithms to improve their search efficiency.

## 4. Material and methods

### 4.1. Experimental set-up

#### 4.1.1. Black Rock Desert set-up

Our experimental set-up consisted of two components, the first being the mobile sensor stack that was carried by a human for collecting odour signals, and the second component included the placement of eight stationary wind sensors for ambient wind measurements around the odour source. The odour source was a propylene gas tank (figure 1*a*) that was mounted with a stationary GPS antenna which sent RTK correction data to the mobile sensor stack (ublox ANN-MB-00-00 Multiband GNSS Antenna connected to a ublox ZED-F9P receiver attached with an RN41 Bluetooth module for pairing with a Dell Laptop running ROS in Ubuntu Linux OS), which together provided high-resolution position with an accuracy close to 1 cm.

The mobile sensor stack in figure 1*b* included a mini photo ionization detector (201A portable, battery powered miniPID photo-ionization detector from Aurora Scientific, operated at its maximum gain settings providing a gas sampling rate of 1250 standard cubic centimetres per minute (SCCM)), a GPS antenna (with the same ublox receiver Bluetooth module as above) to receive accurate location measurements, and an IMU that provided angular velocity measurements. The sensor stack was balanced on a gimbal for stability and ease of carrying. The odour sensor data were collected using a data acquisition board (MC Measurement Computing-USB1608GX), which was connected along with all the other sensors to a computer that was running ROS as middleware and recorded data in real time. Due to the different sampling rates of sensors (wind sensors: 40 Hz, GPS: 5 Hz), the data were linearly interpolated with respect to the rate of the odour sensor which sampled at 200 Hz.

Around the odour source, seven ambient wind sensors (LI-550F TriSonica Mini Wind and Weather Sensor, Trisonica/Li-Cor) were placed 2 m above the ground in a square fashion approximately 30 m away from the source, and one other wind sensor was placed 1 m away from the odour source for accurate measurement of wind speed and direction near the source, as in figure 1*a*,*c*.

#### 4.1.2. Whittell Forest set-up

A similar arrangement of odour source and wind sensors was set up in Whittell Forest to collect odour encounter data under a forest canopy, as in figure 2*a*,*b*. The array of sensors was placed in an L-shaped fashion to better sample the heterogeneous environment (figure 2*c*).

### 4.2. Whiff statistics for trajectory snippets

To understand how a probability distribution of whiff statistics from a short time history is correlated with distance, we randomly selected trajectory snippets of a given length of time. In some cases, our trajectory snippets spanned a wide range of distances. To minimize the artefacts such snippets would create, we first split the datasets from each of our three scenarios into three distance classes (0–5 m, 5–30 m and greater than or equal to 30 m). We randomly selected a total of 500 random trajectory snippets from each distance class, ensuring that each trajectory snippet was unique to a single region.

### 4.3. Calculating whiff frequency

Whiff frequency, wf, is a representation of how often a whiff was encountered within a given duration of time. Following previous applications of wf in related studies, we used a 1 s time window for calculating wf [32]. We converted the time series of odour signals to a binary sequence of delta functions, resulting in a time trace that was zero everywhere except for the first frame of each whiff, which were assigned a value of 1, see figure 4*e*_*i*_. We convolved this sequence of whiff onsets with an exponential filter to determine the whiff frequency

4.1 $$W_{\;freq}(t)=\int_{0}^{t}w(t^{\prime })g(t-t^{\prime })\;dt^{\prime },$$

where *w*(*t*′) is the binary whiff onset signal, *t*^′^ is the time at which we are calculating the wf. The kernel function *g* is defined as follows:

4.2 $$g(t)=\left\{ \begin{array}{cc} \frac{1}{\tau }\;e^{-(1/\tau )(t)}\; & \text{for }t\leq 1\;\\ 0\; & \text{for }t>1\; \end{array}\right.,$$

where *τ* is the decay constant, a measure of how long the previous whiff should be remembered. Based on the whiff data and time constants chosen in [32], we chose *τ* = 2 s (figure 4*d*_*ii*_). To calculate a single wf value for each whiff, we averaged the wf across each individual whiff.

### 4.4. Kalman smoothing

We calculated a continuous estimate of distance to the odour source using a Kalman smoother [47]. We defined a linear and time-invariant discrete dynamical systems model,

4.3 $$\begin{array}{cc} & x_{k+1}=Ax_{k}+Bu_{k}\\ & \;y_{k}=Cx_{k}, \end{array}\}$$

where ***x*** describes two states: the distance to the source and the velocity towards or away from the source. For simplicity, we did not include any control inputs in our model, instead accounting for this modelling uncertainty in our covariance matrix *Q*, resulting in a simple constant velocity dynamic model,

$$A=\left[\begin{array}{cc} 1 & dt\\ 0 & 1 \end{array}\right],\;B=\left[\begin{array}{c} 0\\ 0 \end{array}\right]\;\mathrm{and}\;C=\left[\begin{array}{cc} 1 & 0\\ 0 & 1 \end{array}\right].$$

In a real-world application of an agent searching for an odour source, neither of the states we defined can be directly measured (because the source location is unknown). Thus, for our measurements, we used the following two (imperfect) observer models to estimate a proxy for these states that are then incorporated into the Kalman smoother. We account for the uncertainty resulting from this discrepancy through our measurement covariance matrix *R*. We calculated an estimate of the distance using our combined regression model (equation 2.1) to make an intermittent distance prediction associated with each whiff, based on the look-back time of 10 s. We also compared the performance of distance estimates when using scenario-specific regression models (electronic supplementary material, figure S12). We estimated the velocity towards or away from the source by calculating the sensor’s ground speed component that was parallel to the current ambient wind direction.

For the HWS and LWS scenarios, we defined the measurement and model covariances (*R* and *Q*) to account for the uncertainties in the measurements (*R*) and the dynamic model (*Q*) as follows:

$$R=\left[\begin{array}{cc} 10^{-1} & 0\\ 0 & 10^{-3} \end{array}\right]\;\mathrm{and}\;Q=\left[\begin{array}{cc} 10^{-3} & 0\\ 0 & 10^{-1} \end{array}\right].$$

For the Forest scenario, we were able to reduce the median error with slightly different covariance matrices, which help to account for the increase in variability of wind direction in that dataset

$$R=\left[\begin{array}{cc} 10^{-2} & 0\\ 0 & 10^{-3} \end{array}\right]\;\mathrm{and}\;Q=\left[\begin{array}{cc} 10^{-2} & 0\\ 0 & 1 \end{array}\right].$$

## Data Availability

Data are publicly available at: https://doi.org/10.5061/dryad.2547d7wvr [48]. All analysis code is available on GitHub https://github.com/arunavanag591/odor_analysis [49] and Zenodo https://zenodo.org/doi/10.5281/zenodo.11212243 [50]. The data are provided in electronic supplementary material [51].
